# Unraveling Diabetic Striatopathy: Clinical and Imaging Perspectives

**DOI:** 10.7759/cureus.67105

**Published:** 2024-08-18

**Authors:** Fathima Nilofar, Gowtham Ganapathy, Sharan Bose, Vikrannth V

**Affiliations:** 1 General Medicine, Saveetha Medical College and Hospital, Chennai, IND; 2 Internal Medicine, Saveetha Institute of Medical and Technical Sciences, Chennai, IND

**Keywords:** tetrabenazine, non ketotic hyperglycemia, basal ganglia, hemichorea, hemiballism, diabetes mellitus

## Abstract

Diabetic striatopathy (DS) is an acute hyperkinetic movement disorder arising from non-ketotic hyperglycemia. This condition predominantly affects females and is more common in the elderly, highlighting the interplay between diabetes, striatal pathology, and neurological movement disorders. DS is characterized by involuntary movements, such as hemichorea or hemiballism, and distinctive neuroimaging findings that can be mistaken for more common cerebrovascular events.

In this case report, we describe a 67-year-old female with a history of poorly controlled type 2 diabetes mellitus who presented with the sudden onset of involuntary movements affecting her left upper and lower limbs. Clinical examination and laboratory investigations revealed hyperglycemia without ketosis. Neuroimaging via computed tomography (CT) of the brain identified a hyper density in the right lentiform nucleus, consistent with DS. The patient was treated with vesicular monoamine transporter 2 (VMAT) inhibitors, oral hypoglycemic agents, and insulin, resulting in marked symptom improvement over 10 days. This case underscores the importance of recognizing DS as a differential diagnosis in patients with hyperkinetic movement disorders and hyperglycemia. Proper diagnosis and management, including stringent glycemic control, are crucial for symptom resolution.

## Introduction

Diabetic striatopathy (DS) is a rare but significant neurological complication associated with hyperglycemia in diabetes mellitus (DM). The condition is characterized by hyperkinetic movement disorders such as hemichorea and hemiballism, typically linked to non-ketotic hyperglycemia [1]. The term diabetic striatopathy encompasses various nomenclatures, including hyperglycemic non-ketotic hemichorea/hemiballism and chorea, hyperglycemia, and basal ganglia syndrome [2-4]. First identified as a distinct clinical entity, DS is associated with abnormal metabolic states in the basal ganglia, which are evident in neuroimaging studies [5]. The prevalence of DS is estimated to be one in 100,000, but this figure is likely underestimated due to the frequent misdiagnosis of intracerebral hemorrhage or ischemic stroke [6]. The condition is more prevalent among elderly females, often presenting in the context of poorly controlled type 2 DM with high glycosylated hemoglobin (HHbA1c) levels [7].

Neuroimaging findings, particularly hyperintensity on T1-weighted MRI and hyperdensity on CT, are hallmark features of DS and can aid in distinguishing it from other neurological conditions [8]. DS can manifest as the first indication of diabetes, though it is more commonly associated with long-standing diabetes [9]. Recognizing DS is crucial for clinicians, as the condition is reversible with proper glycemic management and supportive care [10]. Despite its treatability, a lack of awareness about DS can lead to delayed diagnosis and treatment, underscoring the need for heightened clinical suspicion and understanding [11]. This report aims to highlight the clinical presentation, diagnostic challenges, and management strategies for DS through a detailed case study.

## Case presentation

A 67-year-old female with a 10-year history of type 2 diabetes mellitus, characterized by irregular adherence to her medication regimen, presented to the emergency department with the sudden onset of involuntary movements affecting her left upper and lower limbs. These movements had progressively worsened over the past four days, causing significant distress and disability, but were notably relieved during sleep.

On clinical examination, the patient exhibited rapid, dance-like movements involving both the proximal and distal joints of the left limbs, consistent with choreiform movements (video 1). There were no abnormalities detected in the sensory and cerebellar systems, and the rest of the neurological examination was unremarkable.

**Video 1:** Hyperkinetic movement disorder is characterized by hemiballismus-hemichorea of the left upper and lower limbs involving the proximal and distal joints

Laboratory investigations, including a complete blood count (CBC), renal and liver function tests, electrolytes, C-reactive protein (CRP), and erythrocyte sedimentation rate (ESR), were largely within normal limits (Table 1). Urine analysis revealed glycosuria and no evidence of ketosis.

**Table 1:** Laboratory measurements of blood parameters BUN: Blood urea nitrogen, AST: Aspartate transaminase, ALT: Alanine transaminase, CRP: C-reactive protein, ESR: Erythrocyte sedimentation rate, FBS: Fasting blood sugar, PPBS: Post prandial blood sugar, HbA1c: Glycosylated hemoglobin

Parameter	Value	Normal Range
Hemoglobin	13.0	12-15 g/dL
Platelet count	2.88	1.5-4.0 (x 10^6^/mm^3^)
Leukocyte count	8290	4000-10,000 (cells/mm^3^)
BUN	18	7-20 mg/dL
Serum creatinine	1.0	0.6-1.3 mg/dL
AST	25	10-40 U/L
ALT	30	7-56 U/L
Sodium	140	135-145 mmol/L
Potassium	4.0	3.5-5.0 mmol/L
Chloride	103	98-107 mmol/L
Bicarbonate	24	22-29 mmo/L
CRP	0.5	< 1.0 mg/dL
ESR	15	0-20 mm/hr
FBS	358	70-100 mg/dL
PPBS	480	120-140 mg/dL
HbA1c	13.1%	5.4-5.8%

**Figure 1:**
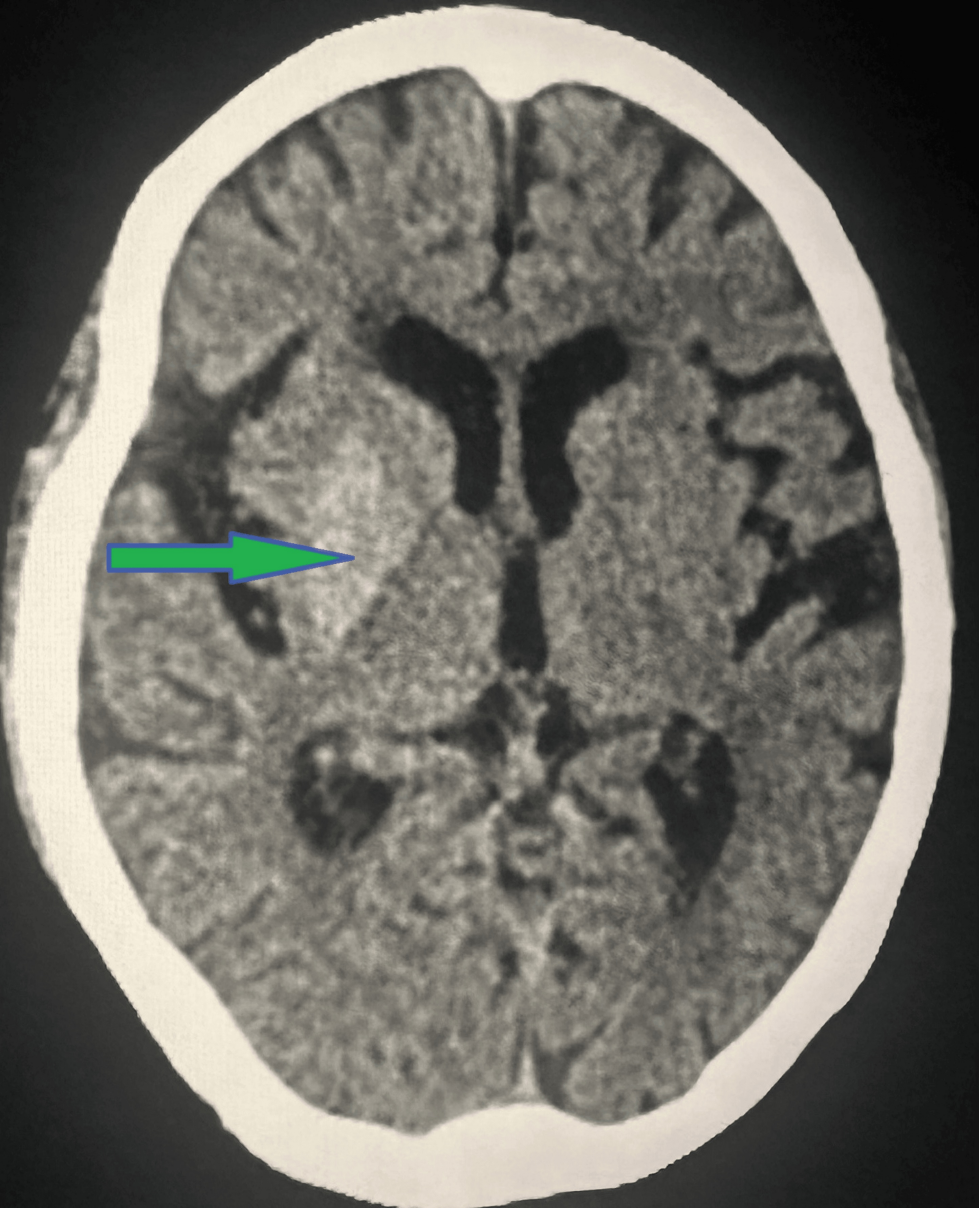
CT brain findings The red arrow shows hyperdensity of HU +35 to +43 noted in the right lentiform nucleus-features suggestive of non-ketotic hyperglycemic hemichorea. CT: Computed tomography, HU: Hounsfield unit

Given the clinical context of poorly controlled diabetes, a computed tomography (CT) scan of the brain was performed, revealing a hyperdensity in the right lentiform nucleus with Hounsfield Units (HU) ranging from +35 to +43, a finding consistent with diabetic striatopathy (hyperglycemic hemichorea/hemiballism) (Figure 1).

The patient’s management was initiated promptly with the administration of vesicular monoamine transporter 2 (VMAT) inhibitors, specifically Tetrabenazine at a starting dose of 25 mg once daily. Concurrently, oral hypoglycemic drugs and insulin therapy were administered to optimize glycemic control. To address the severe involuntary movements, sedatives were provided for symptomatic relief. Given the persistence of the movements, the dosage of tetrabenazine was titrated to 37.5 mg twice daily. Throughout the hospital stay, stringent glycemic control was maintained, resulting in a marked improvement in the patient's symptoms over the course of 10 days. By the time of discharge, the involuntary movements had significantly diminished, allowing for a return to normal daily activities (video 2). The patient was discharged with instructions for continued monitoring and follow-up to ensure ongoing management of both diabetic striatopathy and underlying diabetes mellitus.

**Video 2:** Involuntary movements of the left upper and lower limbs significantly diminished after achieving glycemic control

This case underscores the importance of considering diabetic striatopathy in patients presenting with hyperkinetic movement disorders and hyperglycemia. Early recognition and appropriate management, including glycemic control and symptomatic treatment, are crucial for the resolution of symptoms and the prevention of potential misdiagnosis as well as other neurological conditions.

## Discussion

Diabetic striatopathy (DS) is a rare but significant neurological manifestation of poorly controlled diabetes mellitus, characterized by hyperkinetic movement disorders such as hemichorea and hemiballism. This case highlights the clinical presentation, diagnostic challenges, and management of DS and underscores the importance of differentiating it from other causes of similar movement disorders. In the literature, DS has been reported in various case studies, each contributing to our understanding of this condition.

Herath et al. (2017) described a case of hyperglycemic non-ketotic chorea with rapid radiological resolution, emphasizing the reversible nature of neuroimaging abnormalities following glycemic control [1]. Similarly, Carrion and Carrion (2013) reported a case where DS was initially misdiagnosed as an acute ischemic stroke, underscoring the potential for diagnostic confusion [2]. These cases align with our findings, where the initial presentation and neuroimaging could easily be mistaken for more common cerebrovascular events.

Wang and Song (2015) presented a case with bilateral movements associated with non-ketotic hyperglycemia, highlighting the variability in clinical presentation of DS [3]. In our case, the patient exhibited unilateral movements, consistent with the more typical presentation of DS.

Qi et al. (2012) and Oh et al. (2002) further discussed the characteristic MRI findings of T1 hyperintensity in the basal ganglia, which are pivotal in differentiating DS from other neurological disorders [4,5]. Our patient’s CT scan showing hyperdensity in the right lentiform nucleus corroborates these findings.

Ryan et al. (2018) reviewed cases of hyperglycemic chorea/ballism over 15 years, noting that DS is often underdiagnosed due to its rarity and the unfamiliarity of many clinicians with the condition [7]. This highlights the importance of awareness and consideration of DS in differential diagnosis when patients present with hyperkinetic movement disorders and hyperglycemia. The management of DS primarily involves strict glycemic control and symptomatic treatment.

Ondo (2011) and Son et al. (2017) demonstrated that the resolution of hyperglycemia leads to a significant improvement in symptoms, similar to our patient, who showed marked improvement with insulin therapy and VMAT inhibitors [6,12]. The use of tetrabenazine, as employed in our case, has been effective in managing hyperkinetic symptoms, as also noted by Sitburana and Ondo (2006) [13].

Bizet et al. (2014) and Chua et al. (2020) have emphasized the need for a high index of suspicion for DS, especially in elderly diabetic patients presenting with movement disorders [10,8]. The reversible nature of both the clinical symptoms and neuroimaging abnormalities, along with proper treatment, reinforces the importance of early diagnosis and intervention.

DS is a rare but clinically significant condition that can be effectively managed with timely recognition and appropriate treatment. This case, along with the reviewed literature, underscores the importance of considering DS in patients with hyperkinetic movement disorders and hyperglycemia to avoid misdiagnosis and ensure proper management.

## Conclusions

Diabetic striatopathy (DS) is a rare hyperkinetic movement disorder arising from non-ketotic hyperglycemia in diabetes mellitus patients. The condition's hallmark is the presence of involuntary movements like hemichorea or hemiballism, often misdiagnosed due to their rarity and overlapping symptoms with other neurological conditions. Accurate diagnosis relies on distinctive neuroimaging findings, such as hyperdensity in the basal ganglia on CT scans and hyperintensity on T1-weighted MRI. Effective management of DS requires prompt recognition and stringent glycemic control, which can lead to significant improvement and reversal of symptoms. This case emphasizes the necessity for clinicians to be aware of DS to avoid misdiagnosis and ensure appropriate treatment. The successful resolution of symptoms in our patients with the use of VMAT inhibitors and insulin therapy underscores the importance of targeted therapeutic interventions. Ongoing research and increased case reporting are vital to further elucidate the pathophysiology of DS and develop standardized diagnostic and treatment protocols. Multidisciplinary collaboration is crucial for optimizing patient care. Recognizing DS as a differential diagnosis in hyperglycemic patients presenting with movement disorders can greatly enhance clinical outcomes and patient quality of life.
